# Indicators of integrating oral health care within universal health coverage and general health care in low-, middle-, and high-income countries: a scoping review

**DOI:** 10.1186/s12903-023-02906-2

**Published:** 2023-04-29

**Authors:** Yassaman Karimi Jashni, Fatemeh Emari, Martin Morris, Paul Allison

**Affiliations:** 1 Faculty of Dental Medicine and Oral Health Sciences, McGill University, Montréal, Canada; 2 Faculty of Medicine and Health Sciences, McGill University, Montréal, Canada; 3 Schulich Library of Physical Sciences, Life Sciences and Engineering, McGill University, Montréal, Canada

**Keywords:** Universal Health Coverage, Oral health care, Oral health, Indicators, WHO, Monitoring framework

## Abstract

**Background:**

The World Health Organization (WHO) has recently devoted special attention to oral health and oral health care recommending the latter becoming part of universal health coverage (UHC) so as to reduce oral health inequalities across the globe. In this context, as countries consider acting on this recommendation, it is essential to develop a monitoring framework to measure the progress of integrating oral health/health care into UHC. This study aimed to identify existing measures in the literature that could be used to indicate oral health/health care integration within UHC across a range of low-, middle- and high-income countries.

**Methods:**

A scoping review was conducted by searching MEDLINE via Ovid, CINAHL, and Ovid Global Health databases. There were no quality or publication date restrictions in the search strategy. An initial search by an academic librarian was followed by the independent reviewing of all identified articles by two authors for inclusion or exclusion based on the relevance of the work in the articles to the review topic. The included articles were all published in English. Articles concerning which the reviewers disagreed on inclusion or exclusion were reviewed by a third author, and subsequent discussion resulted in agreement on which articles were to be included and excluded. The included articles were reviewed to identify relevant indicators and the results were descriptively mapped using a simple frequency count of the indicators.

**Results:**

The 83 included articles included work from a wide range of 32 countries and were published between 1995 and 2021. The review identified 54 indicators divided into 15 categories. The most frequently reported indicators were in the following categories: dental service utilization, oral health status, cost/service/population coverage, finances, health facility access, and workforce and human resources. This study was limited by the databases searched and the use of English-language publications only.

**Conclusions:**

This scoping review identified 54 indicators in a wide range of 15 categories of indicators that have the potential to be used to evaluate the integration of oral health/health care into UHC across a wide range of countries.

**Supplementary Information:**

The online version contains supplementary material available at 10.1186/s12903-023-02906-2.

## Background

In 2015, the Member States of the United Nations (UN) set Universal Health Coverage (UHC) as one of the targets to be achieved by 2030 as a part of the Sustainable Development Goals (SDGs) agreement (Target 3.8) [1]. This target was reasserted in the United Nations General Assembly High-Level Meeting on UHC in 2019 [2]. UHC has been defined as “the desired outcome of health system performance, whereby all people who need the full spectrum of health services (that is, promotion, prevention, treatment, rehabilitation, and palliation) receive them according to need, without resulting in hardship (including possible impoverishment caused by out-of-pocket payments) because of any associated health care costs” [3].

Countries are making progress towards UHC, and governments have set different strategies to move towards this goal [3]. These strategies follow the main elements of UHC: access, coverage, service quality, and financial protection [3]. Moreover, a direct correlation exists between achieving progress towards UHC and progressing in additional health goals [2]. This is because sustainable improvements in UHC enhance populations’ health and economic well-being [1, 2]. UHC is a guiding principle for providing health services for a wide range of people and health problems, including oral health problems. Therefore, oral health care services could, and indeed should, also be defined as part of UHC principles to provide accessible and affordable services to a community.

The 2017 Global Burden of Disease (GBD) study reports indicated that around 3.5 billion people are affected by oral diseases globally, including untreated caries in permanent teeth as the most common non-communicable disease (NCD) [4]. Severe periodontal diseases and oral cancer are also oral health conditions that raise the incidence rate of populations’ oral disease levels in different parts of the world [4]. Furthermore, treatment costs, out-of-pocket payments, and lack of access to oral health care services additionally affect populations’ oral health [5]. Therefore, in 2019, oral health was included in the WHO Political Declaration on UHC, with the aim of promoting accessible and affordable oral health care services throughout the world [2]. Additionally, oral care services are included in the UHC Compendium, which is a database that assists countries in achieving progress towards UHC [6].

Along the same lines of the UHC strategies, in 2020, a The Lancet Commission on Global Oral Health [7] was established to reflect on different plans and policies for the improvement of oral health and the revision of dental health care services globally to make oral health and oral health care more accessible for all people, particularly those with the highest burden of disease and the poorest access to care.

In 2021, the WHO published specific resolutions on oral health during their 148th session, and in the World Health Assembly resolution WHA74.5, explicitly mentioned repositioning oral health as part of the global health agenda in the context of the UHC [8, 9]. The resolution addressed delivering oral health services as part of UHC and drafting a global strategy for implementing the most efficient and effective interventions in public oral health systems across the world. Accompanying this, the WHO engaged to develop a global oral health strategy and accompanying action plan setting a framework to assess the progress of oral health care integration into UHC [8, 9].

Around the same time but as a separate initiative, the World Dental Federation (Federation Dentaire Internationale; FDI), which comprises national associations of dentists across the globe, published the “FDI Vision 2030” report addressing the assimilation of good quality, essential oral health services into the general medical health care system in every country by the year 2030 [10]. This vision stated that the combination of oral and general person-centred health care results in more effective prevention and management of oral diseases [10].

Taken together, these separate initiatives of a Lancet Commission, the WHO and the FDI indicate that there is a strong world view to integrate oral health care into general health care and into UHC.

To evaluate UHC implementation in a country, recommendations have been made on setting up a monitoring framework based on various elements [3]. The suggested elements may differ from one country to another because economic, social, health care system, and other factors differ [3]. The two main indicators being used to measure the progress of UHC as SDG target 3.8 are essential health services coverage and financial risk protection against service costs (financial hardship) [11, 12]. Based on these two indicators, the World Bank and the WHO built a framework (referred to as the *WHO/WB framework* in the rest of this article) to monitor the implementation and progress of UHC in health systems [13]. This WHO/WB framework includes a selection of fourteen key indicators classified in four main categories: (i) reproductive, maternal, new-born, and child health; (ii) infectious diseases; (iii) non-communicable diseases; and (iv) service capacity and access [14]. Most of the 14 key indicators in these categories assess factors that are recognized to be common across many countries [14]. However, among the suggested indicators, there are no measures that involve or are specific to oral health and/or dental care. If progress is to be made in the aforementioned vision of integrating oral health care into general health care and UHC, it is not possible to evaluate progress within and across nations without clear, agreed-upon indicators. Setting up a monitoring framework including such indicators is crucial to evaluate the progress towards these goals in any community, whether national or global. Furthermore, given the global outlook of the aforementioned organizations, it is crucial to identify indicators of the integration of oral health care into general health care and UHC for a broad range of countries. If we are to address this issue from a global perspective, consideration needs to be given to low-, middle- and high-income countries, as well as those that already have UHC or not and multiple other factors that will differ across countries.

In this context, the aim of this project was to identify indicators that that have the potential to demonstrate the extent to which oral health care is integrated within general health care and UHC across a broad range of low-, middle-, and high-income countries.

## Methods

A scoping review “provides a preliminary assessment of the potential size and scope of available research literature. It aims to identify the nature and extent of research evidence (usually including ongoing research)” [15]. This scoping review aimed to identify indicators relevant to the integration of oral health care into UHC and general health care. It was conducted based on Arksey and O’Malley’s methodological framework for scoping reviews [16]. Based on the research question, an academic medical librarian (MM) developed a search strategy for exploring related literature in the MEDLINE via Ovid database. The search strategy was converted for CINAHL and Ovid Global Health databases in advance (Table 1). Following the aforementioned definition of a scoping review focusing on research literature, we decided not to search grey literature. The databases were searched using Medical Subject Headings (MeSH) or their equivalent, keywords, truncations, and adjacency operators; these terms were combined using standard Boolean operators. Universal health coverage, universal health insurance, oral health, and dental health services were defined as key concepts for the search strategy. The searches were carried out on May 13, 2021 and updated on September 16, 2021. No language, publication date, geographic limit, or quality restrictions (including primary study articles, reviews, meeting abstracts, conference proceedings, book chapter reviews/articles, and commentaries) were applied.

**Table 1:** Search strategy (developed for searching Medline)

1. exp universal health care/
2. ((universal adj2 (health or coverage or insurance)) or (social* adj2 (coverage or insurance))).tw,kf.
3. (essential adj2 (healthcare or health care)).tw,kf.
4. exp Universal Health Insurance/
5. (essential adj2 (healthcare or health care)).tw,kf.
6. or/1–5
7. exp Dentistry/
8. exp Oral Health/
9. exp Stomatognathic Diseases/
10. exp Dental Health Services/
11. (dentist* or denturist* or ((dental or oral) adj3 (health or care or surgeon? or office? or clinic? or assistant? or nurse? or hygien* or practitioner? or professional? or auxiliar*))).tw,kf.
12. (dentist* or endodont* or orthodonti* or periodont* or prosthodont* or oropharyng* or jaw or jaws or mandibular or maxillofacial or mandible* or maxilla* or tooth or teeth or odontolog* or tongue* or glossal or buccal or palatal or palate or palates or labial or lip or lips or gingiva* or gingiviti* or halitosis or bad breath or DMF).tw,kf.
13. or/7–12
14.6 and 13

**Fig. 1:**
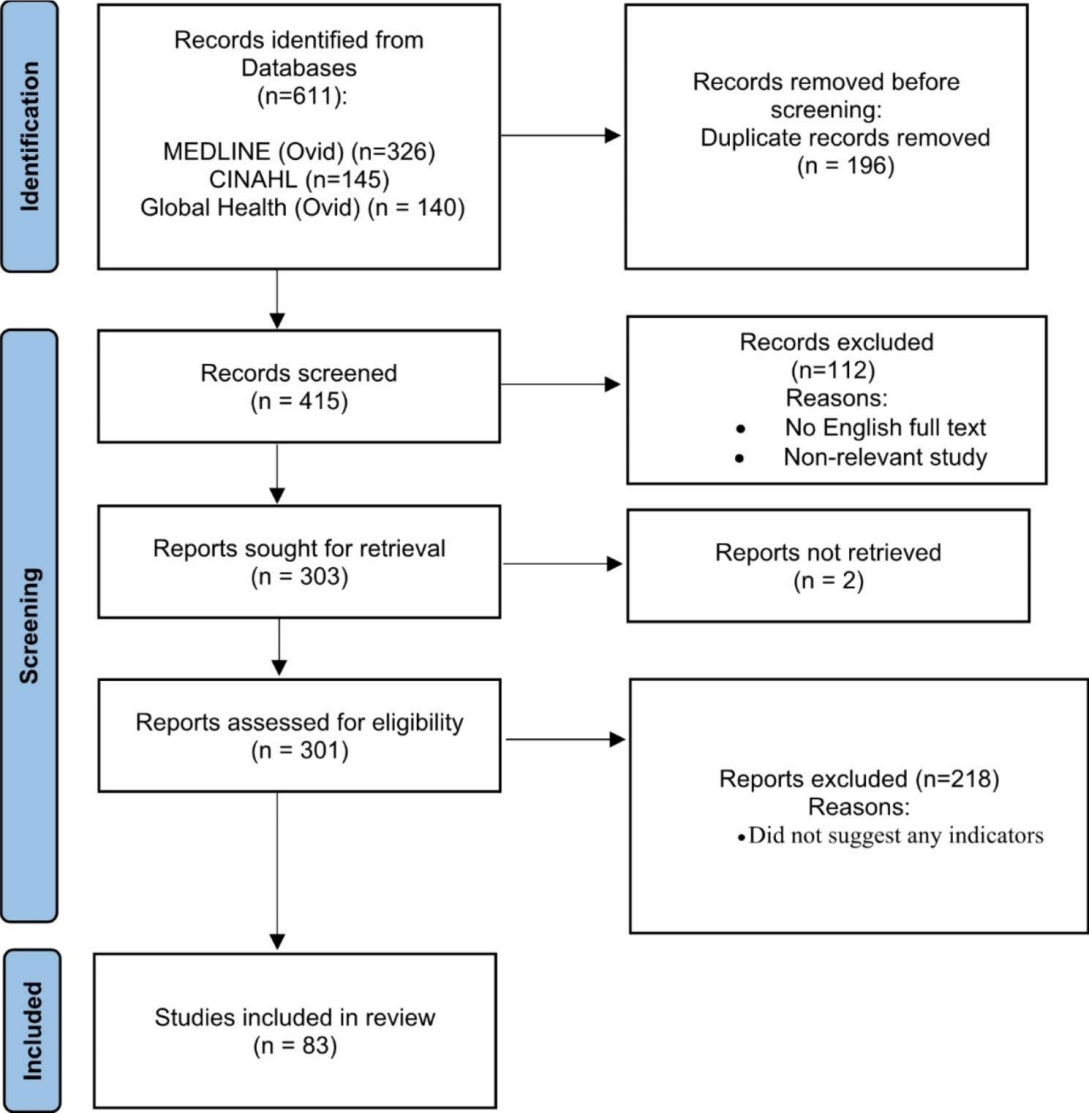
Article selection procedure for the scoping review PRISMA 2020 version

Six hundred and eleven (611) document records were identified through searching the above-mentioned databases. The exclusion of the duplicates resulted in 415 records. Two team members screened the 415 articles’ titles and abstracts independently to explore the documents potentially relevant to the aim of the study and the research question. Following this primary title/abstract screening, 114 articles were removed from the search list as they were either unrelated to the primary concept of the study or in languages other than English. The result was that 301 articles were fully reviewed by two team members independently. The goal of this project was to identify indicators that could be used to measure a health care system’s progress towards integrating oral health care into UHC. In view of this, we included articles with indicators of service utilization, insurance coverage, care expenses, health facility access, health status, health care providers, knowledge of health, availability and acceptability of services, need and demand for dental care, health policies, fluoride, oral hygiene, and Infection control. The search strategy shown in Table 1 shows the precise terms used as the inclusion keywords. Articles that had no suggestions on indicators were excluded. Eighty-three articles were retained after the application of these inclusion and exclusion criteria. Articles that the two reviewers disagreed on were reviewed by a third author, and agreement with the original reviewers reached on articles to be included. The article assessment process is displayed in a Preferred Reporting Items for Systematic Reviews and Meta-Analyses (PRISMA) flowchart (see Fig. 1. Article selection procedure for the scoping review PRISMA 2020 version) [17, 18].

Once the articles to be included were agreed, a data extraction was developed and piloted. The two reviewers appraised ten articles and evaluated their results to ensure the process’s reliability by identifying differences and reaching agreement on how to report these differences through the process. The data extracted from each article were: the articles’ DOI/web address; publication year; country of origin; and a detailed description of the indicator(s). This process was performed independently by two reviewers. Relevant indicators were then extracted from included articles. Ultimately, the data were collected and descriptively mapped based on a simple frequency count of the indicators.

No ethical approval was needed as this study was based on already published data.

## Results

Reviewing the 83 included articles resulted in distinct categories of indicators relevant to the topic of interest. Included articles were published in English and covered a wide range of 32 low-, middle- and high-income countries, although work from only one low-income country was identified among these countries. The publication dates spanned 1995 to 2021.

This scoping review identified a total of 54 different indicators. In particular, 34 indicators were classified under 14 main categories, describing indicator definitions, specific terms used to represent indictors, and variations by which indicators were measured in the studies. Six indicators were not assigned to any categories, and 14 indicators did not have specific definitions or clear examples. Different potential sources of data collection have been suggested in the table of results. These sources were: individuals in the population, dental professionals and government staff including public health officials.

Among the 14 defined categories, “dental service utilization” and “oral health status” were the categories with the highest numbers of potential indicators and/or the categories with indicators repeated most often. In the selected articles, “dental service utilization” included two indicators that were mentioned a total of 65 times in various formats among a wide range of high- to low-income countries. In addition, this category encompasses the most frequently monitored indicator, which is “Visiting a dentist in the past 12 months”. In the category “oral health status”, eight indicators were stated 58 times in various countries.

“Coverage” was a category with three indicators: “cost coverage”, “service coverage”, and “population coverage”. These indicators were reported 26 times in various formats among countries with diverse socioeconomic statuses. Financial coverage and out-of-pocket costs are indicated by “cost coverage”. “Service coverage” refers to the type of services covered by a plan and the term “population coverage” refers to which groups in the population are covered by a plan.

Another three indicators that were classified as the “finances” category were: “costs for the provider”, “costs/expenses for the patient”, and “funding sources”, which were stated 22 times in several countries. “Health facility access” included three indicators named “geographic access”, “health facility access”, and “home care”. These indicators were mentioned 21 times in different countries.

The remaining categories of the identified indicators were “workforce”, “knowledge of oral health”, “fluoride”, “oral hygiene”, “availability and acceptability of services”, “need and demand for dental care”, “diet”, “policies” and “infection control”. The indicators classified under these categories were reported fewer times and in a limited number of countries compared to the aforementioned indicators. The results have been comprehensively demonstrated in Table 2.

**Table 2:** Table of results

Factor	Indicator	Source of data	Specific terms*	Variation**	Countries or regions used	Number of times used	References
Dental service utilization	Consultation /visit (professional/ dental care facility) over certain period	Individuals +/or government sources	Visit in last 3 months (3)	As an access indicator By number of visits By age By frequency By Time since last visit By purpose of visit (need only, check-up, treatment) By reason (costs, waiting lists, travel difficulties) By service type By number of days	Low-income: Burkina Faso Middle-income: Brazil, China, Colombia, Malaysia, Mauritius, Nigeria, Peru, Thailand, Turkey High-income: Australia, Canada, Chile, Denmark, Estonia, Europe, Finland, Israel, Ireland, Japan, Spain, Sweden, Taiwan, UK, US	65	[19–70]
			Visit over last 6 months (1)				
			Visit in last 12 months (23)				
			Visit last 2 years (1)				
			Visit in last 5 years (1)				
			Visit in 5 + years (1)				
			Home visit (dentist/dental hygienist) (1)				
			Last dental appointment/visit (3)				
			Preventive dental visit (1)				
			Visit specialist (1)				
			Receive care in last 2 years (1)				
			Number of dental treatments annually (1)				
			First visit (1)				
			Visit dentist /Dental care visit (6)				
			Dental/oral health check-ups (2)				
			Visit only for emergency (1)				
			Foregone dental care in last 12 months (1)				
			Never seen a dentist in life (1)				
			Having a regular dentist (1)				
			Days spent on dental care in a year (1)				
			Dentalcare/dental services utilization in last 12 months (2)				
			Utilization of primary oral health service (1)				
	Type of treatment obtained	Individuals +/or government sources	Extraction (3)	By type			
			Type of treatment obtained (4)				
			Fillings (2)				
			Prescription of medication (1)				
Coverage	Cost coverage	Individuals +/or public health or government sources	Insurance coverage (3)	By type (e.g., public/private/mutual oral care coverage)	Middle-income: Brazil, China, Colombia, Nigeria, Senegal, Thailand, Turkey High-income: Europe, Israel, Japan, US	26	[27, 35, 42, 43, 46, 49, 67, 71–80]
			Health Insurance system (1)				
			Insurance scheme (1)				
			Healthcare Coverage type (1)				
			Social health insurance (1)				
			Cost coverage package (2)				
	Service coverage	Individuals +/or public health or government sources	Service coverage (2)				
			Dental coverage (4)				
			Oral health coverage (2)				
			Comprehensive services (1)				
			Major services (1)				
			Basic services (1)				
			Preventive services (1)				
			Adoption of prevention and oral health promotion (1)				
			The extent of oral health services in the UHC benefit packages (1)				
	Population coverage	Individuals +/or public health or government sources	Population coverage (3)				
Finances	Costs for provider	Provider representative	Cost of material/equipment (1)	By payment type (out of the pocket) By ability to pay Costs prevented receiving treatment	Middle-income: Brazil, Colombia, Nigeria, Senegal, Thailand, Turkey High-income: Australia, Israel, Japan, Taiwan	22	[27, 35, 48, 49, 54, 57, 67, 68, 71, 72, 74, 78–83]
	Costs/expenses for patient	Individuals +/or public health or government sources	Cost of treatment/oral health care (3)				
			Oral Health/dental care expenditures (4)				
			Ability to pay (4)				
			Out of pocket payment (6)				
			Affordability of services (1)				
	Funding sources	public health or government representative	Government (2)				
			Donors (1)				
Health facility access	Geographic access	public health or government sources	Health facility geographic location (4)	By geographic location of facility/distance By physical availability of the facility By travel time By service access rate By availability By accessibility	Middle-income: Nigeria, Thailand, Turkey High-income: Australia, Europe, Japan, Taiwan	21	[22, 35, 48, 54, 57, 67, 68, 72, 76, 78, 79, 81–84]
		Individuals	Travel time (2)				
			Outreach to rural/underserved populations (1)				
	Health facility access	Individuals +/or public health or government sources	Health centre number/dental facility (3)				
			Availability of service (2)				
			Type of facility utilized (3)				
			Access to dental care service (3)				
			Person’s ability to obtain necessary care (1)				
	Home care	Individuals +/or public health or government sources	Home oral rehabilitation services (1)				
			Dental Home Care Management (1)				
Oral health status	DMFT	Profession +/or public health or government sources	DMFT/dmft (9)	By score	Low-income: Burkina Faso Middle-income: Brazil, China, Colombia, Nigeria, Serbia, Thailand High-income: Australia, Canada, Denmark, England, Finland, Germany, Israel, Japan, Korea, Netherlands, US	58	[20, 22, 25, 26, 28, 40, 41, 43, 46, 57, 62, 67, 68, 71, 72, 74, 78, 85–94]
			dmfs (1)				
	Missing teeth	Profession +/or public health or government sources	Missing teeth (6)	By number			
			Tooth loss (4)				
			Edentulism (2)				
			Number of natural teeth in adults (2)				
			Survival of permanent teeth (1)				
	Pain in teeth	Individual or profession or public health	Pain/toothache (2)	By severity			
	Periodontal condition	Profession +/or public health or government sources	Periodontal condition/disease (6)	-			
			Attachment loss > = 4 mm (1)				
	Caries	Profession +/or public health or government sources	Untreated caries/caries lesion (4)	By age (in children)			
			Dental caries (4)				
			Fillings with secondary caries (1)				
			Caries free teeth (1)				
			Untreated tooth decay (1)				
	Oral mucosa disease	Profession +/or public health or government sources	Oral mucosa disease (1)	-			
	Craniomandibular dysfunction	Profession +/or public health or government sources	Craniomandibular dysfunction (1)	-			
	Oral health condition	Individual or profession or public health	Dental fluorosis (1)	-			
			Use of dentures/denture wearing (2)				
			Chewing ability (2)				
			Poor oral health (condition) (1)				
			Tetracycline-stained teeth (1)				
			Oral health assessment (1)				
			Self-reported oral health (2)				
			Disability caused by severe tooth loss (1)				
Workforce	Dental workforce	Profession +/or public health or government sources	Dental workforce/Human resource number (4)	By availability By number in population (between urban and rural areas) By ratio	Middle-income: China, Nigeria, Senegal High-income: Australia, Europe, Ireland, Japan, Taiwan	11	[27, 43, 51, 57, 67, 72, 76, 78, 80, 82, 95]
			Human resource availability (1)				
			Shortage of trained dental personnel (1)				
			Qualified dentalcare staff (1)				
			Dentist/population ratio (2)				
			Geographic distribution of health providers (1)				
	Attitude of health provider	Profession	Attitude of health provider (1)				
Knowledge	Awareness of oral health	Individual or profession or public health	Awareness/knowledge of oral health (4)	By rate (improved) By education status (socioeconomic factor)	Middle-income: Nigeria, Senegal, Thailand, Turkey High-income: Germany, Japan	9	[23, 35, 47, 67, 78, 80, 82, 93]
			Population education (2)				
			Health education and information (1)				
			Information on oral health care (1)				
			Oral health literacy (1)				
Fluoride	Water fluoridation	Public health or government	Fluoridated water exposure (1)	By exposure As collective prophylaxis	Low-income: Burkina Faso Middle-income: Brazil, China High-income: Canada, Germany, Israel, Japan	7	[26, 62, 71, 78, 91–93]
			Fluoridation of the water supply (2)				
			Fluoride intake (1)				
	Fluoride prophylaxis	Individual or profession or public health	Fluoridated table salt (1)				
			Topical fluoride (1)				
			Fluoride toothpaste (1)				
Oral hygiene	Oral hygiene	Individual	Practicing interproximal cleaning (1)	By habit type (cleaning, chewing sticks, brushing, flossing) By frequency	Low-income: Burkina Faso Middle-income: China, Thailand High-income: Canada, Finland, Germany, Japan, US	7	[19, 23, 26, 40, 62, 78, 92]
			Hygiene habit (6)				
Availability and acceptability of service	Waiting time	Individual or profession or public health	Waiting time for appointment (2)	By waiting time By speed	Middle-income: Colombia, Thailand High-income: Australia, Finland, US	6	[40, 49, 57, 79, 81, 96]
			Waiting room time (1)				
			Satisfaction with last treatment period (1)				
			Speed of services and referral system (1)				
	Acceptability/satisfaction	Individual	Acceptability of service (1)				
Need and demand for dental care	Unmet needs	Individual or public health	No unmet need for oral health services in the prior 12 months (1)	By unmet needs and oral condition	Middle-income: China, Ghana, India High-income: Australia	5	[27, 57, 81, 97]
			Reasons for unmet needs (1)				
			Annual incidence of unmet oral health needs (1)				
	Perceived need	Individual	Perceived need for treatment (1)				
	Demand	Individual or profession	Health demands (1)				
Diet	Sugar consumption	Individual or public health	Sugar consumption (1)	-	Low-income: Burkina Faso Middle-income: China, Thailand High-income: Germany, Japan	5	[23, 26, 78, 92, 93]
			Drink sugar-sweetened beverage (1)				
	Diet	Individual or public health	Eating healthy food (1)				
			Dietary habits (2)				
Policies	Government policies	Public health or government	Government policies for oral health (1)	-	Middle-income: Nigeria	2	[67, 82]
			Policies for oral health (1)				
Infection control	Infection control resources	Profession or public health	Infection control resources (1)	-	Middle-income: Nigeria	1	[82]
Other	Other		Health status (1)	-	Middle-income: China, Colombia, Thailand	6	[23, 27, 43, 49, 72]
			Contact oral health services with the broader health system (1)				
			Transport and appropriate technologies (1)				
			Effective dental education system (1)				
			The proportion of primary care, services, promotion, and prevention (1)				
			Social support about oral health (towards periodontal status) (1)				
**Possible indicators which do not have clear examples**: (numbers in brackets are reference numbers) - “Engaging the local population in integrating oral health into universal health coverage.” [98] - “Educating the society on oral care delivery model.” [99] - “Oral health team should acquire a thorough understanding of the importance that social determinants play in oral as well as general health.” [100] - “Dentists and the oral health team should engage in partnership with communities to help them better understand and tackle the social, economic, and environmental factors that determine oral health and increase inequalities.” [100] - “Dentists and the oral health team should engage with colleagues such as primary health care professionals (cross-sectoral partnerships).” [100] - “Dentists should become advocates for health, particularly oral health, with their patients and the wider community.” [100] - “Advancement of the population’s knowledge, attitudes, and skills towards oral health.” [101] - “Expansion of support, and development of cohesiveness and partnerships in achieving oral health.” [101] - “Organizational change such as policy, regulatory, and strategic directions.” [101] - “Workforce development such as integrating dental public health discipline in professional learning programs.” [101] - “Resource allocation to achieve new/reorient available resources for health promotion and prevention.” [101] - “Impowering leadership skills for advocacy, lobbying, and awareness raising.” [101] - -“Developing partnership, shared goals, and planning oral health integrated programs with the community and between different organizations for capacity building.” [101]							

*Numbers in parentheses represent the frequency of each indicator

** The ‘variation’ column describes how were the indicators been measured in the studies

## Discussion

In recent years, special attention has been directed by a range of international organizations and groups to oral health care and its integration into UHC. To ensure progress is made in this integration process, it is important to have a monitoring framework incorporating relevant indicators. This framework should be adaptable to monitor progress in a range of low-, middle-and high-income countries. It should also be simple, practical, and comprehensive to cover all relevant oral health care domains. Currently, there is no such framework available to monitor the implementation of oral health care into the UHC, although this is being developed as part of the preparation of a global oral health action plan by the WHO. This framework and the aforementioned WHO plan need to use relevant indicators to track how the integration process is progressing in countries across the world. This scoping review has identified oral health care indicators that could be used as part of a global monitoring framework for oral health care integration into UHC and general health care.

Different frameworks are being used to monitor UHC development in a range of countries and health systems. For instance, the *WHO/WB framework* has been used as the main framework in many countries, although these countries adjusted the framework to measure the progress of UHC in the desired health care scopes, based on the needs of their populations. These country-specific frameworks have many similarities but also have some differences in accordance with the different regions of the world in which they are being used [102, 103].

In the context of the review reported in this paper, it is interesting to note that we can see how most of the indicators we identified fit into these existing frameworks developed for a broad range of health services beyond oral health care. For instance, “visit an oral health care facility or an oral health professional” was the most frequently reported indicator in our scoping review and it is similar to an indicator such as the “number of antenatal and postnatal visits”, which was used in the *WHO/WB framework* and its country-specific versions used in a range of countries such as Bangladesh, Iraq, South Africa, and India [14, 102–105]. Similarly, “Oral health status” indicators were the second most frequent set of indicators found in the literature. They cover a wide range of indicators from “DMFT” to “craniomandibular dysfunction” and “oral health condition”. The *WHO/WB framework* focuses on NCD health status indicators such as “blood pressure”, “blood glucose”, and “cervical cancer prevalence” to monitor the general health status of the population [14, 106]. Similar to that, other frameworks used various health status indicators as treatment indicators [103, 107–109]. Therefore, oral health status indicators could fit into the existing frameworks with the same aim.

On top of these examples, “Cost-, service-, and population-coverage” were coverage indicators reported numerously in the oral health care literature, and they are essentially the same as “coverage of the health services” and “financial protection”, which were the two main components used in the *WHO/WB framework* [13]. Furthermore, “service coverage” was the key indicator for developing the *WHO/WB framework* to follow UHC implementation in health systems [3, 14]. These three coverage indicators are the three main components of UHC that could be used as leading indicators for monitoring progress in integrating dental care within UHC [110]. They enable us to understand the progress towards the target of achieving UHC in different health systems.

As well as these examples of how oral health indicators mirror those of general health care indicators, additional ones can also be provided for indicators in “health facility access” and “workforce” categories. That said, it is important to note that some oral indicators we identified do not have general health care peers. “Fluoride exposure” and “oral hygiene” were oral health-related indicators that will not fit into the existing monitoring frameworks such as the *WHO/WB framework*, and the aforementioned regional and country modified frameworks. Indicators related to the “need and demand for dental care”, “policies”, and “infection control” domains were also found in the oral health care literature that could not be found in the existing monitoring frameworks. Among other suggested indicators, these indicators might be used as future measures to assess the UHC progress, although some could not be measured as system variables (such as “diet”).

Among the indicators identified in this review, there are a number that are relatively straightforward to collect, while others are more complex to both define and collect. Among the former group are indicators that have been used many times and can be collected relatively easily to monitor progress. For example, the “proportion of the population visiting a dental health care professional once a year” and “insurance coverage”. These are relatively straightforward to define and collect for instance through system administrative data or through self-complete surveys. However, there are a number of indicators we identified whose definition is unclear and may vary across countries, such as “awareness of oral health” and “need and demand for dental care”. These indicators illustrate well both the possibility of different definitions and the subsequently different means and so the feasibility of collecting the data. For example is the need for oral health care defined by clinicians (requiring a clinical examination), by people in the population (requiring a self-complete survey) or through administrative data (e.g. as defined by having had no consultation over a period of x years)? Added to the complexity of collecting such data is the expense, particularly for performing clinical examinations.

This scoping review was conducted to identify potential oral health care-related indicators for monitoring the implementation of oral health care into UHC and general health care. The results of this study were limited as we only searched a few databases, and in particular, we did not search the so-called “grey literature” of government survey reports on oral health and oral health care, which contain many examples of the types of indicators we were searching for. Furthermore, the limited number of databases restricted the scope of this research in terms of identifying indicators in various sociocultural contexts. In addition, the list of countries that the identified indicators were drawn from featured just one low-income country. This finding may be attributed to the previously discussed limitations of the study or may suggest significant constraints related to scarcity of resources in low-income countries. Another limitation was that we included only publications written in English. Although the results covered a broad range of oral health care categories, some areas could be missing. Consulting professional experts in the field could help transcend this limitation. Indeed, we believe the indicators identified by this search are an initial step in identifying a collection of indicators relevant to a wide range of countries, which could be complemented by others that are more specific to countries in particular regions of the world or low-, middle- and high-income groups.

## Conclusions

There is a need for a monitoring framework to evaluate the progress of oral health care integration into UHC and general health care. This scoping review identified indicators in a wide range of oral health care domains relevant to the integration of oral health care into UHC and general health care. Many of these indicators were relevant to all forms of health care, including oral health care, whereas some were more specific for developing the oral health care monitoring framework. While it is possible that we missed some oral health care indicators in our review, when comparing our results with those of the *WHO/WB framework*, it seems we have included all the categories of indicators. Further studies, as well as interviews with experts, could be conducted with the aim of finding more indicators and choosing the most relevant ones to achieve a consensus on creating a practical and comprehensive monitoring framework for oral health care integration into UHC and general health care.

## Electronic supplementary material

Below is the link to the electronic supplementary material.

Supplementary Material 1

## Data Availability

The datasets used and/or analysed during the current study are available from the corresponding author on reasonable request.

## List of abbreviations

WHO: World Health Organization
UHC: Universal Health Coverage
UN: United Nations
SDGs: Sustainable Development Goals
NCD: Non-communicable Disease
FDI: World Dental Federation (Federation Dentaire Internationale)
PRISMA: Preferred Reporting Items for Systematic Reviews and Meta-Analyses
