# Supplementary material for: Indicators of integrating oral health care within universal health coverage and general health care in low-, middle-, and high-income countries: a scoping review
Source: BMC Oral Health. 2023 Apr 29;23:251. doi: 10.1186/s12903-023-02906-2 (PMC10149008; doi:10.1186/s12903-023-02906-2)
Supplement: Supplementary file 1 — Supplementary Material 1 [file 12903_2023_2906_MOESM1_ESM.docx]

**Additional table1.** Exact wording for each indicator.

| **Factor:** | **Indicator*:** |
| --- | --- |
| Consultation/ dental service utilization | **Consultation/visit over certain period:**  Visit to a dentist in the past 3 months [1]  Attended in any oral health service 3 months prior to the survey [2]  Visited a dentist in the previous 3 months [3]  Visits to a dentist over the last 6 months [4]  Visiting a dental professional past year [5]  Self‑reported usage of healthcare services in the last year (dentist Consultation) [6]  Attended a dentist in the previous year [7]  Percentages of people who had at least one visit to oral health services during last year [8]  Visiting a dentist in the past 12 months [9]  Dentist visit in 12 months [10]  Dental attendance during the past 12 month (latest dental treatment visits, Dental check-up visits) [11]  Visited the dentist in the previous year [12]  Attendance for dental check-ups in past 12 months [13]  Visits to dental care facility in one year [14]  Dentist visits in the past 12 month [15]  Visit to a dentist in the previous 12 months [16]  Dental care use in the past year (visit) [17]  Dental attendance in the past year [18]  Use of dental services in 12 months [19]  Number of visits for dental care in the past 12 months [20]  Consultation with a dentist in the past year [21]  Visit dentist in 12 months [22]  Access to/ receive service from Dentist within past year [23]  Use of care (dental visit in last year) [24]  Number of visits during a calendar year [25]  A time since the last visit of under 12 months [26]  Consultation with a dentist during the year before the interview [27]  Mean number of visits to dentist in the previous two years [28]  Regular dental visits carried out for dental check-ups over the past 5 years [29]  Visited dentist 5+ years ago, Last visited for a problem [30]  Dental Home Visits (dentist)/ Home Visits by Dental Hygienists [31]  Last dental appointment [32]  Last dental visit [33]  Time since last visit to dental care [34]  Health care utilization (preventive dentist visit) [35]  Visit to a specialist [35]  Receiving care during the 2-year period of study interviews [36]  Number of dental treatments annually [37]  First visit of the refugees as the service utilization [38]  Number of visits for dental care [39]  Visit dentist (6 monthly, yearly, on need only) [40]  Taken to a Dentist (visit dentist) [41]  Dental care visits [42]  Dental visits [43]  Dental visits (Age at First Dental Visit (<2, 2-5, >5), Reason for First Dental Visit, Frequency of Dental Visits) [44]  Regular oral health check-ups [45]  Uptake of regular health check-ups (dental check-up) [46]  Visiting a dentist only for emergencies [9]  Foregone dental care in last 12 months [47]  Never seen a dentist in their life [7]  Having a regular dentist [48]  Number of days spent on dental care services during the year [43]  Dental care use had occurred within the last 12 months [49]  Dental care utilisation (visit in last 12 month) [50]  Utilization of primary oral health services [51]  **Type of treatment obtained:**  Dental extractions [52]  A reported extraction at the last visit [26]  Received extractions <12 month [30]  Type of treatment obtained [20]  Treatment obtained [39]  Treatment obtained [53]  Type of treatment [38]  Dental fillings [52]  Received fillings <12 month [30]  Medicine supply [35] |
| Coverage | **Cost coverage:**  Insurance ownership [41]  Insurance coverage [19]  Health insurance [52]  Health insurance system [54]  Insurance scheme [55]  Type of healthcare coverage [56]  Social health insurance [35]  Share of total costs covered [57]  Cost coverage package [58]  **Service coverage:**  Service coverage [58]  Services covered [57]  Dental insurance coverage [50]  Dental insurance coverage [36]  Dental insurance coverage [18]  Dental coverage [59]  Oral health coverage [60]  Mutual oral care coverage [61]  Major services [57]  Basic services [57]  Comprehensive services [57]  Preventive services [57]  Adoption of prevention and oral health promotion [62]  The extent of oral health services in the UHC benefit packages [8]  **Population coverage:**  Population coverage [8]  Population coverage [58]  Who is covered [57] |
| Finances | **Costs for provider:**  Cost of raw materials/Cost of dental equipment and materials [63]  **Costs for patient:**  Treatment costs [62]  Oral health care cost [61]  **Cost** prevented receive treatment/ Avoided/delayed due to cost [30]  Financing oral health services (current health expenditure on oral health services per capita) [8]  Health expenditure [54]  Dental care expenditure (household spending on health) [59]  The ability of consumers to pay [63]  Poverty (low income/ ability to pay) [64]  Higher income [56]  Income [41]  Financial sources such as out-of-pocket payments [8]  Out-of-pocket payment [20]  Health expenditures (out-of-pocket expenditure) [35]  Cost and payment (out of pocket payment) [52]  Healthcare expenditure [60]  Out-of-pocket payments [39]  Out-of-pocket payments for oral healthcare [53]  Affordability of services [65]  **Funding sources:**  government funding [63]  governments [8]  donors [8] |
| Health facility access | **Geographic access:**  Access to oral healthcare services (geographic location/ distance from the city) [52]  Geographical distance [65]  Geographic inequalities in access [30]  Geographical location of the provider [63]  Travel times to the nearest public dental clinic [66]  Long patient travel time [52]  Outreach to rural and other underserved populations [62]  **Health facility access:**  Location and number of oral health facilities [63]  Number of dental facilities [54]  More public health centers [56]  Physical Availability of services [65]  Physical availability of services [58]  Type of facility utilized [20]  Type of facility utilised [39]  Type of facility where utilized [53]  Increased accessibility to healthcare services [64]  Access problems [41]  Ease of current access to professional care (Better transportation) [56]  Person’s ability to obtain necessary care [58]  **Home care:**  Home oral rehabilitation services [31]  Dental Home Care Management [31] |
| Oral health status | **DMFT:**  DMFT [60]  DMFT [67]  DMFT index [68]  DMFT [69]  DMFT [54]  DMFT score [70]  Decayed, filled or missing teeth due to caries (dmft) [71]  Decayed, missing, or filled teeth (DMFT) [72]  Decayed/missing/filled teeth level [59]  dmfs [44]  **Missing teeth:**  Missing Teeth [31]  Number of missing teeth [32]  Missing teeth [67]  Missing Teeth (MT) [68]  Number of missing teeth [73]  Self-reported number of missing teeth [34]  Tooth loss (as tooth extraction due to decay or gum disease by a dentist in the past 12 months) [9]  Total tooth loss [74]  Tooth loss [72]  Tooth loss and edentulousness [68]  Edentulism [75]  Edentulousness (no natural teeth) [73]  Number of teeth in adults [54]  Self-reported number of natural teeth [29]  Survival of Teeth [31]  **Pain in teeth:**  Experienced a toothache <12 month [30]  Severe pain in tooth [52]  **Periodontal condition:**  Oral health status (periodontal diseases) [19]  Periodontal disease [72]  Periodontal diseases [67]  Periodontitis condition [74]  Periodontal diseases [68]  Periodontal Disease [31]  Attachment loss >=4mm [7]  **Caries:**  Untreated caries [74]  Having ≥3 teeth with untreated carious lesions [73]  Caries lesions [71]  Untreated caries [7]  Caries experience [70]  Dental Caries [31]  Prevalence of dental caries in children [54]  Caries [19]  Fillings with secondary caries and caries sequels [71]  Caries-free primary dentition [68]  Epidemiological (Untreated tooth decay) [62]  **Oral mucosa disease:**  Oral mucosal diseases [68]  **Craniomandibular:**  Craniomandibular dysfunction [68]  **Oral health condition:**  Dental fluorosis [19]  Use of dentures [17]  Denture wearing [50]  Chewing ability [50]  Chewing ability [17]  Self-reported poor oral health [76]  Tetracycline-stained teeth [19]  Oral health assessment [32]  Self-reported oral health [9]  Self-assessed oral health status [53]  Disability caused by severe tooth loss [62] |
| Workforce | **Dental workforce:**  Human resource number [52]  The number of oral health workforce per 10 000 population [8]  Supply of dentists per 100 000 population between urban and rural areas [30]  Dental workforce number [54]  Human resource availability [63]  Shortages of appropriately trained dental personnel [62]  Qualified dental care staff [61]  Health resources (distribution of dental service workforce (dentist to population ratio)) [19]  Dentist/population ratio [37]  Geographic distribution of health providers (e.g., dentists) [77]  **Attitude of health provider:**  Attitude of the provider [58] |
| Knowledge | Consumer awareness of oral health [63]  Improved awareness of oral health [54]  Knowledge of respondents on oral health [51]  Oral health awareness (know enough about dental care) [52]  Information on oral health care [61]  Educational status [52]  Education of the parent [41]  Health education and information [68]  Oral health Literacy (skills toward health information and services, knowledge and understanding of health information, communication, Self-management, media literacy, decision-making) [45] |
| Fluoride | **Water fluoridation:**  Exposure to fluoridated water [44]  Fluoridation of the water supply [59]  Water fluoridation [72]  Fluoride intake [67]  **Fluoride prophylaxis:**  Collective prophylaxis (fluoridated table salt) [68]  Topical fluoride [54]  Fluoride toothpaste [7] |
| Oral hygiene | The frequency of practicing interproximal cleaning [5]  Cleaning or brushing teeth [45]  Adults cleaned their teeth less often than once per day [7]  Brushing behaviour [54]  Tooth brushing frequency [34]  Oral hygiene practices [67]  Oral hygiene habits (Dental flossing, Frequency of toothbrushing) [44] |
| Healthcare utilization/ acceptability of services | **Waiting time:**  Health service quality (waiting days) [35]  Waited >6 month for appointment [30]  Waiting room time [78]  Satisfaction with the last dental treatment period [34]  Faster health services and referral systems [56]  **Acceptability:**  Acceptability of services (family condition, cultural factors) [65] |
| Need and demands | **Unmet need:**  No unmet need for oral health services in the prior 12 months [79]  Reasons for unmet need [8]  Annual incidence of unmet oral health needs [8]  **Perceived need:**  Perceived needs for treatment [30]  **Demand:**  Health demands [65] |
| Diet  Sugar consumption | **Sugar consumption:**  Sugar consumption [54]  Drink a sugar-sweetened beverage [7]  **Diet**:  Eating healthy food [45]  Dietary habits [67]  Diet [68] |
| Policies | Government policies [52]  Policies for oral health [63] |
| Infection control | Infection control resources [63] |

* Numbers in the brackets are reference numbers.

**References:**

1. Garrido-Cumbrera M, Borrell C, Palencia L, Espelt A, Rodriguez-Sanz M, Pasarin MI, et al. Social class inequalities in the utilization of health care and preventive services in Spain, a country with a national health system. Int J Health Serv. 2010;40(3):525-42.

2. Hernandez-Vasquez A, Bendezu-Quispe G, Azanedo D, Santero M. Use of oral health care services in Peru: trends of socio-economic inequalities before and after the implementation of Universal Health Assurance. BMC Oral Health. 2019;19(1):39.

3. Palència L, Espelt A, Rodríguez-Sanz M, B Rocha K, Isabel Pasarín M, Borrell C, et al. Trends in social class inequalities in the use of health care services within the Spanish National Health System, 1993-2006. Eur J Health Econ. 2013;14(2):211-9.

4. Habicht J, Kunst AE. Social inequalities in health care services utilisation after eight years of health care reforms: a cross-sectional study of Estonia, 1999. Soc Sci Med. 2005;60(4):777-87.

5. Yuqing Z, Leveille SG, Ling S, Camhi SM, Zhang Y, Shi L. Disparities in Preventive Oral Health Care and Periodontal Health Among Adults With Diabetes. Prev Chronic Dis. 2021;18:1-9.

6. Galvao TF, Tiguman GMB, Pereira Nunes B, Correia da Silva AT, Tolentino Silva M. Continuity of Primary Care in the Brazilian Amazon: A Cross-Sectional Population-Based Study. Int J Prev Med. 2021;12:57.

7. Clauss A, Sie A, Zabre P, Schmoll J, Sauerborn R, Listl S. Population-Based Prevalence of Oral Conditions as a Basis for Planning Community-Based Interventions: An Epidemiological Study From Rural Burkina Faso. Front. 2021;9:697498.

8. Agrasuta V, Thumbuntu T, Karawekpanyawong R, Panichkriangkrai W, Viriyathorn S, Reeponmaha T, et al. Progressive realisation of universal access to oral health services: what evidence is needed? BMJ glob. 2021;6(7):07.

9. Zivkovic N, Aldossri M, Gomaa N, Farmer JW, Singhal S, Quinonez C, et al. Providing dental insurance can positively impact oral health outcomes in Ontario. BMC Health Serv Res. 2020;20(1):124.

10. Pulok MH, Gool Kv, Hall J. Horizontal inequity in the utilisation of healthcare services in Australia. Health Policy. 2020;124(11):1263-71.

11. Cooray U, Aida J, Watt RG, Tsakos G, Heilmann A, Kato H, et al. Effect of Copayment on Dental Visits: A Regression Discontinuity Analysis. J Dent Res. 2020;99(12):1356-62.

12. Pilotto LM, Celeste RK. The relationship between private health plans and use of medical and dental health services in the Brazilian health system. Cienc. 2019;24(7):2727-36.

13. Kino S, Bernabe E, Sabbah W. Social Inequalities in Use of Preventive Dental and Medical Services among Adults in European Countries. Int J Environ Res Public Health. 2019;16(23):22.

14. Bakar NSA, Manual A, Hamid JA. Socioeconomic Status Affecting Inequity of Healthcare Utilisation in Malaysia. Malays. 2019;26(4):79-85.

15. Dehmoobadsharifabadi A, Singhal S, Quinonez CR. Impact of public dental care spending and insurance coverage on utilization disparities among Canadian jurisdictions. J Public Health Dent. 2018;78(4):346-51.

16. Kiil A, Arendt JN. The effect of complementary private health insurance on the use of health care services. Int J Health Econ Manag. 2017;17(1):1-27.

17. Murakami K, Hashimoto H. Wealth-related versus income-related inequalities in dental care use under universal public coverage: a panel data analysis of the Japanese Study of Aging and Retirement. BMC Public Health. 2016;16:24.

18. Manski R, Moeller J, Chen H, Widstrom E, Listl S. Disparity in dental attendance among older adult populations: a comparative analysis across selected European countries and the USA. Int Dent J. 2016;66(1):36-48.

19. Liu J, Zhang SS, Zheng SG, Xu T, Si Y. Oral Health Status and Oral Health Care Model in China. Chin J Dent Res. 2016;19(4):207-15.

20. Somkotra T. Experience of socioeconomic-related inequality in dental care utilization among Thai elderly under universal coverage. Geriatr Gerontol Int. 2013;13(2):298-306.

21. Lebrun LA. Effects of length of stay and language proficiency on health care experiences among immigrants in Canada and the United States. Soc Sci Med. 2012;74(7):1062-72.

22. Allin S. Does Equity in Healthcare Use Vary across Canadian Provinces? Healthc Policy. 2008;3(4):83-99.

23. Lasser KE, Himmelstein DU, Woolhandler S. Access to care, health status, and health disparities in the United States and Canada: results of a cross-national population-based survey. Am J Public Health. 2006;96(7):1300-7.

24. Jones JA. Financing and reimbursement of elders' oral health care: lessons from the present, opportunities for the future. J Dent Educ. 2005;69(9):1022-31.

25. Suominen-Taipale AL, Widstrom E. A longitudinal study of young Finnish adults' use of subsidized, private sector dental care, 1986-1997. Community Dent Oral Epidemiol. 2000;28(5):365-72.

26. Roberts-Thomson K, Brennan DS, Spencer AJ. Social inequality in the use and comprehensiveness of dental services. Aust J Public Health. 1995;19(1):80-5.

27. Rajmil L, Borrell C, Starfield B, Fernandez E, Serra V, Schiaffino A, et al. The quality of care and influence of double health care coverage in Catalonia (Spain). Arch Dis Child. 2000;83(3):211-4.

28. Davidson T, Rohlin M, Hultin M, Jemt T, Nilner K, Sunnegardh-Gronberg K, et al. Reimbursement systems influence prosthodontic treatment of adult patients. Acta Odontol Scand. 2015;73(6):414-20.

29. Petersen PE, Davidsen M, Rosendahl Jensen H, Ekholm O, Illemann Christensen A. Trends in dentate status and preventive dental visits of the adult population in Denmark over 30 years (1987-2017). Eur J Oral Sci. 2021:e12809.

30. Schwarz E. Access to oral health care - an Australian perspective. Community Dent Oral Epidemiol. 2006;34(3):225-31.

31. Okamoto E. Japan's Dental Care Facing Population Aging: How Universal Coverage Responds to the Changing Needs of the Elderly. Int J Environ Res Public Health. 2021;18(17):04.

32. Galvao MHR, Medeiros AA, Roncalli AG. Contextual and individual factors associated with public dental services utilisation in Brazil: A multilevel analysis. PLoS ONE. 2021;16(7):e0254310.

33. Boccolini CS, de Souza Junior PR. Inequities in Healthcare utilization: results of the Brazilian National Health Survey, 2013. Intern. 2016;15(1):150.

34. Raittio E, Aromaa A, Kiiskinen U, Helminen S, Suominen AL. Income-related inequality in perceived oral health among adult Finns before and after a major dental subsidization reform. Acta Odontol Scand. 2016;74(5):348-54.

35. Ruiz Gomez F, Zapata Jaramillo T, Garavito Beltran L. Colombian health care system: results on equity for five health dimensions, 2003-2008. Rev Panam Salud Publica. 2013;33(2):107-15, 6 p preceding

36. Kreider B, Manski RJ, Moeller J, Pepper J. The effect of dental insurance on the use of dental care for older adults: a partial identification analysis. Health Econ. 2015;24(7):840-58.

37. Guiney H, Felicia P, Whelton H, Woods N. Analysis of a payments database reveals trends in dental treatment provision. J Dent Res. 2013;92(7 Suppl):63S-9S.

38. Zimmerman M, Bornstein R, Martinsson T. Utilization of dental services in refugees in Sweden 1975-1985. Community Dent Oral Epidemiol. 1995;23(2):95-9.

39. Somkotra T, Vachirarojpisan T. Inequality in dental care utilisation among Thai children: evidence from Thailand where universal coverage has been achieved. Int Dent J. 2009;59(6):349-57.

40. Paurobally N, Kruger E, Tennant M. Are diabetes and dental care providers in the Republic of Mauritius advising patients about the importance of oral health in diabetes management? International Journal of Dental Hygiene. 2021;19(2):184-92.

41. Caner A, Karaoglan D, Yasar G. Utilization of health-care services by young children: The aftermath of the Turkish Health Transformation Program. Int J Health Plann Manage. 2018;12:12.

42. Wen PC, Lee CB, Chang YH, Ku LE, Li CY. Demographic and rural-urban variations in dental service utilization in Taiwan. Rural Remote Health. 2017;17(3):4161.

43. Nishide A, Fujita M, Sato Y, Nagashima K, Takahashi S, Hata A. Income-Related Inequalities in Access to Dental Care Services in Japan. Int J Environ Res Public Health. 2017;14(5):12.

44. Ismail AI, Sohn W. The impact of universal access to dental care on disparities in caries experience in children. J Am Dent Assoc. 2001;132(3):295-303.

45. Khamrin P, Boonyathee S, Bootsikeaw S, Ong-Artborirak P, Seangpraw K. Factors Associated with Health Literacy, Self-Efficacy, Social Support, and Oral Health Care Behaviors Among Elderly in Northern Border Community Thailand. Clin Interv Aging. 2021;16:1427-37.

46. Sabates R, Feinstein L. Do income effects mask social and behavioural factors when looking at universal health care provision? Int J Public Health. 2008;53(1):23-30.

47. Elstad JI. Dental care coverage and income-related inequalities in foregone dental care in Europe during the great recession. Community Dent Oral Epidemiol. 2017;45(4):296-302.

48. Graham MA, Tomar SL, Logan HL. Trends. Perceived social status, language and identified dental home among Hispanics in Florida. Journal of the American Dental Association (JADA) (American Dental Association). 2005;136(11):1572-82.

49. Listl S. Countries With Public Dental Care Coverage Have Lower Social Inequalities in the Use of Dental Services Than Countries Without Such Coverage. Journal of Evidence-Based Dental Practice. 2015;15(1):41-2.

50. Sgan-Cohen H, Livny A, Listl S. Dental health among older Israeli adults: is this a reflection of a medical care model inadequately addressing oral health? Int Dent J. 2015;65(1):49-56.

51. Adeleke CA, Eke JH. FACTORS AFFECTING UTILIZATION OF PRIMARY ORAL HEALTH CARE SERVICES: A STUDY OF SELECTED POPULATION IN IBADAN. West African Journal of Nursing. 2014;25(2):75-88.

52. Uguru N, Onwujekwe O, Uguru CC, Ogu UU. Achieving universal health coverage in Nigeria: the dilemma of accessing dental care in Enugu state, Nigeria, a mixed methods study. Heliyon. 2021;7(1):e05977.

53. Somkotra T, Detsomboonrat P. Is there equity in oral healthcare utilization: experience after achieving Universal Coverage. Community Dent Oral Epidemiol. 2009;37(1):85-96.

54. Zaitsu T, Saito T, Kawaguchi Y. The Oral Healthcare System in Japan. Healthcare (Basel). 2018;6(3):10.

55. Srinarupat J, Oshiro A, Zaitsu T, Prasertsom P, Niyomsilp K, Kawaguchi Y, et al. Inequalities in Periodontal Disease According to Insurance Schemes in Thailand. Int J Environ Res Public Health. 2021;18(11):01.

56. Kerdpon D, Jantharapattana K, Sriplung H. Factors related to diagnostic delay of oral squamous cell carcinoma in southern Thailand: Revisited. Oral Dis. 2018;24(3):347-54.

57. Allin S, Farmer J, Quinonez C, Peckham A, Marchildon G, Panteli D, et al. Do health systems cover the mouth? Comparing dental care coverage for older adults in eight jurisdictions. Health Policy. 2020;124(9):998-1007.

58. Palm W, Webb E, Hernandez-Quevedo C, Scarpetti G, Lessof S, Siciliani L, et al. Gaps in coverage and access in the European Union. Health Policy. 2021;125(3):341-50.

59. Rosen B, Waitzberg R, Merkur S. Israel: health system review: Health Systems in Transition (European Observatory on Health Systems and Policies); 2015. 17(6):xxv + 212 pp. many ref.; 2015.

60. Nascimento AC, Moyses ST, Werneck RI, Moyses SJ. Oral health in the context of primary care in Brazil. Int Dent J. 2013;63(5):237-43.

61. Diop M, Kanoute A, Diouf M, Ndiaye AD, Lo CMM, Faye D, et al. The role of health insurance in the coverage of oral health care in Senegal. Journal of Public Health in Africa. 2018;9(3):174-8.

62. Mathur MR, Williams DM, Reddy KS, Watt RG. Universal health coverage: a unique policy opportunity for oral health. J Dent Res. 2015;94(3 Suppl):3S-5S.

63. Uguru N, Onwujekwe O, Ogu UU, Uguru C. Access to Oral health care: a focus on dental caries treatment provision in Enugu Nigeria. BMC Oral Health. 2020;20(1):145.

64. Wang F, Wang J, Hung Y. Universal health insurance, health inequality and oral cancer in Taiwan. PLoS ONE. 2018;13(10).

65. Ghanbarzadegan A, Balasubramanian M, Luzzi L, Brennan D, Bastani P. Inequality in dental services: a scoping review on the role of access toward achieving universal health coverage in oral health. BMC Oral Health. 2021;21(1):404.

66. Jean G, Kruger E, Tennant M. Universal access to oral health care for Australian children: comparison of travel times to public dental services at consecutive census dates as an indicator of progressive realisation. Aust J Prim Health. 2020;26(2):109-16.

67. Saekel R. China's oral care system in transition: lessons to be learned from Germany. Int J Oral Sci. 2010;2(3):158-76.

68. Ziller S, Micheelis W, Oesterreich D, Reich E. Goals for oral health in Germany 2020. Int Dent J. 2006;56(1):29-32.

69. Hyman JJ, Reid BC, Mongeau SW, York AK. The military oral health care system as a model for eliminating disparities in oral health. Journal of the American Dental Association (JADA) (American Dental Association). 2006;137(3):372-8.

70. Verlinden DA, Reijneveld SA, Lanting CI, Wouwe JPv, Schuller AA. Socio-economic inequality in oral health in childhood to young adulthood, despite full dental coverage. Eur J Oral Sci. 2019;127(3):248-53.

71. Markovic D, Soldatovic I, Vukovic R, Peric T, Campus GG, Vukovic A. How Much Country Economy Influences ECC Profile in Serbian Children-A Macro-Level Factor Analysis. Front. 2019;7:285.

72. Pucca GA, Jr., Gabriel M, Araujo MEd, Almeida FCSd. Ten years of a national oral health policy in Brazil innovation, boldness, and numerous challenges. J Dent Res. 2015;94(10):1333-7.

73. Guarnizo-Herreno CC, Watt RG, Garzon-Orjuela N, Suarez-Zuniga E, Tsakos G. Health insurance and education: major contributors to oral health inequalities in Colombia. J Epidemiol Community Health. 2019;73(8):737-44.

74. Bernabe E, Marcenes W, Hernandez CR, Bailey J, Abreu LG, Alipour V, et al. Global, Regional, and National Levels and Trends in Burden of Oral Conditions from 1990 to 2017: A Systematic Analysis for the Global Burden of Disease 2017 Study. J Dent Res. 2020;99(4):362-73.

75. Ito K, Cable N, Yamamoto T, Suzuki K, Kondo K, Osaka K, et al. Wider Dental Care Coverage Associated with Lower Oral Health Inequalities: A Comparison Study between Japan and England. Int J Environ Res Public Health. 2020;17(15):31.

76. Kim N, Kawachi I. Did the expansion of insurance coverage for oral health reduce self-reported oral health inequalities in Korea? Results of repeated cross-sectional analysis, 2007-2015. J Epidemiol. 2020;30(12):537-41.

77. Yang CH, Huang YT, Hsueh YS. Redistributive effects of the National Health Insurance on physicians in Taiwan: a natural experiment time series study. Intern. 2013;12:13.

78. Kashner TM. Research issues related to oral health expenditures and financing oral health care for the aging veteran. Med Care. 1995;33(11 Suppl):NS90-105.

79. Kailembo A, Preet R, Stewart Williams J. Socioeconomic inequality in self-reported unmet need for oral health services in adults aged 50 years and over in China, Ghana, and India. Intern. 2018;17(1):99.
